# Zika virus disrupts molecular fingerprinting of human neurospheres

**DOI:** 10.1038/srep40780

**Published:** 2017-01-23

**Authors:** Patricia P. Garcez, Juliana Minardi Nascimento, Janaina Mota de Vasconcelos, Rodrigo Madeiro da Costa, Rodrigo Delvecchio, Pablo Trindade, Erick Correia Loiola, Luiza M. Higa, Juliana S. Cassoli, Gabriela Vitória, Patricia C. Sequeira, Jaroslaw Sochacki, Renato S. Aguiar, Hellen Thais Fuzii, Ana M. Bispo de Filippis, João Lídio da Silva Gonçalves Vianez Júnior, Amilcar Tanuri, Daniel Martins-de-Souza, Stevens K. Rehen

**Affiliations:** 1 D’Or Institute for Research and Education (IDOR), Rio de Janeiro, Brazil; 2 Institute of Biomedical Sciences, Federal University of Rio de Janeiro, Rio de Janeiro, Brazil; 3 Institute of Biology, Department of Biochemistry and Tissue Biology, University of Campinas (UNICAMP), Campinas, Brazil; 4 Center for Technological Innovation, Evandro Chagas Institute, Belém, Brazil; 5 Institute of Biology, Federal University of Rio de Janeiro, Rio de Janeiro, Brazil; 6 Institute Oswaldo Cruz, FIOCRUZ, Rio de Janeiro, Brazil; 7 Federal University of Pará, Belém, Brazil

## Abstract

Zika virus (ZIKV) has been associated with microcephaly and other brain abnormalities; however, the molecular consequences of ZIKV to human brain development are still not fully understood. Here we describe alterations in human neurospheres derived from induced pluripotent stem (iPS) cells infected with the strain of Zika virus that is circulating in Brazil. Combining proteomics and mRNA transcriptional profiling, over 500 proteins and genes associated with the Brazilian ZIKV infection were found to be differentially expressed. These genes and proteins provide an interactome map, which indicates that ZIKV controls the expression of RNA processing bodies, miRNA biogenesis and splicing factors required for self-replication. It also suggests that impairments in the molecular pathways underpinning cell cycle and neuronal differentiation are caused by ZIKV. These results point to biological mechanisms implicated in brain malformations, which are important to further the understanding of ZIKV infection and can be exploited as therapeutic potential targets to mitigate it.

Primary Microcephaly is a rare brain malformation characterized by a reduction of the cephalic perimeter. The etiology of microcephaly varies from genetic abnormalities to external agents such as the STORCH factors–Syphilis, *Toxoplasma gondii*, Rubella, Cytomegalovirus and Herpes virus infections^1^.

An increased number of cases of microcephaly associated with Zika virus (ZIKV) has been reported in Brazil and elsewhere^2^,^3^. ZIKV belongs to the *Flaviviridae* family, which also comprises other important pathogens such as Hepatitis C virus (HCV), West Nile virus (WNV), Japanese encephalitis virus (JEV) and dengue virus (DENV). Since the outbreak of ZIKV-associated microcephaly was observed, the cellular effects of ZIKV infection were rapidly explored. ZIKV alters cell cycle and triggers caspase-mediated cell death in iPS-derived neural progenitors^4^. It reduces the growth of brain organoids^5^ and impairs neuronal differentiation *in vitro*^6^. Recent studies using mouse models also confirmed the association between ZIKV infection and brain malformations^7^,^8^. RNA data from microcephalic embryonic mice^8^, human fibroblasts^9^ and neural progenitors^4^ showed deregulation of many individual genes related to viral response. The molecular pathways associated with ZIKV self-replication and its relation to the failure of central nervous system growth is yet to be discovered.

Here we examine the interactome map of proteins and genes altered by ZIKV infection. Combining large scale, state of the art transcriptome and proteome analysis, we identified molecular pathways associated with the Brazilian ZIKV infection in human neurospheres. We show that ZIKV alters the molecular fingerprint of neural stem cells by activating responses to viral replication, DNA damage targets, cell cycle arrest, apoptosis, as well as the downregulation of neurogenic programs. These results shed light on potential molecular mechanisms implicated in brain malformations as a result of congenital ZIKV infection.

## Results

We have focused on studying the effects of the Brazilian strain of ZIKV. Using distance matrix analysis, we compared the viral envelope, NS1, NS2A, NS2B and NS3 of the Brazilian isolate to those of several African ZIKV strains, as well as to the Asian ZIKV strains. The analysis showed that the Brazilian isolate (BR_ZIKV_AB_ES) was at the minimum 12.2% divergent from each of the analyzed African strains. In contrast, the greatest sequence divergence between the Brazilian isolate and each of the Asian strains isolated from *Homo sapiens* was 1.9% (Supplementary Fig. S1A and Supplementary Table S1). This clearly shows that Brazilian ZIKV fragments sequence-matched the Asian ZIKV strains. This result is in agreement with previous observations that the ZIKV circulating in Brazil is 97–100% similar to the ZIKV isolated in Asia^2^. Data illustrating the conservation among the genes is also provided (Supplementary Fig. S2). By performing RT-PCR with primers specific for ZIKV, Dengue and Chikungunya, we confirmed that the BR_ZIKV_AB_ES isolate contained only ZIKV and was not contaminated with other viruses commonly found in Brazil.

To unveil molecular pathways potentially associated with microcephaly, we analyzed the molecular effects of ZIKV on human stem cell-derived neurospheres. Neural stem cells derived from induced pluripotent stem (iPS) cells were exposed to ZIKV (MOI 0.025) for two hours. Free-floating differentiation allowed the formation of neurospheres, which are enriched in Nestin and Sox2 positive cells. In addition to self-renewal, these cells also differentiate into Tuj1 positive neurons and S100 positive glial cells (Supplementary Fig. S3).

**Figure 1:**
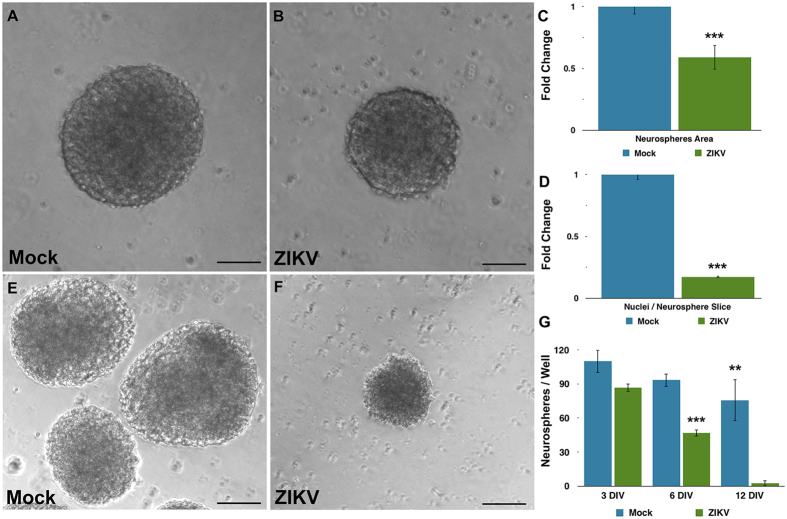
Reduction in growth of human neurospheres infected with Zika virus. (**A,B**) Brightfield photomicrographs of Mock- and ZIKV-infected neurospheres. Bar graphs showing a reduction of neurospheres area (**C**) and in numbers of nuclei per neurosphere area (**D**) on ZIKV-infected experimental group. (**E**) Time-course of neurospheres viability of Mock- and ZIKV-infected experimental groups. Data presented as mean ± SD, n = 4, Student’s t-test, **p < 0.01. (**F,G**) Brightfield photomicrographs of Mock- and ZIKV-infected neurospheres at day 12. Calibration Bar: 100 μm.

ZIKV infection impaired the growth of neurospheres over time, although cells were still viable. Three days after infection, both mock (exposed to Vero cells’ supernatant) and ZIKV-infected cultures formed neurospheres (Fig. 1A,B). However, infected neurospheres were significantly smaller in comparison to mock neurospheres (Fig. 1C). In addition, DAPI staining suggests a positive correlation between reduced-sized neurospheres and nuclei counting (Fig. 1D).

Although neurosphere size was altered three days post-infection, there was no statistical difference in the number of neurospheres in the ZIKV infected group compared to mock. However, after six days *in vitro*, the number of ZIKV-infected neurospheres was reduced by 50% (2791 ± 178.1 versus 1653 ± 160, mean ± SEM). Finally, after twelve days of ZIKV infection *in vitro*, neurospheres were entirely depleted (Fig. 1E–G). In order to determine the effect of ZIKV directly in the neural progenitors of the neurospheres, we infected isolated neural stem cell cultures containing Sox2 expressing cells, as well as radial glia-like cultures containing Pax6, Vimentin and p-Vimentin positive cells (Supplementary Fig. S4). Similar to ZIKV-infected neurospheres, both cell cultures displayed cell death after six days *in vitro* (Supplementary Figs S5 and S6).

**Figure 2:**
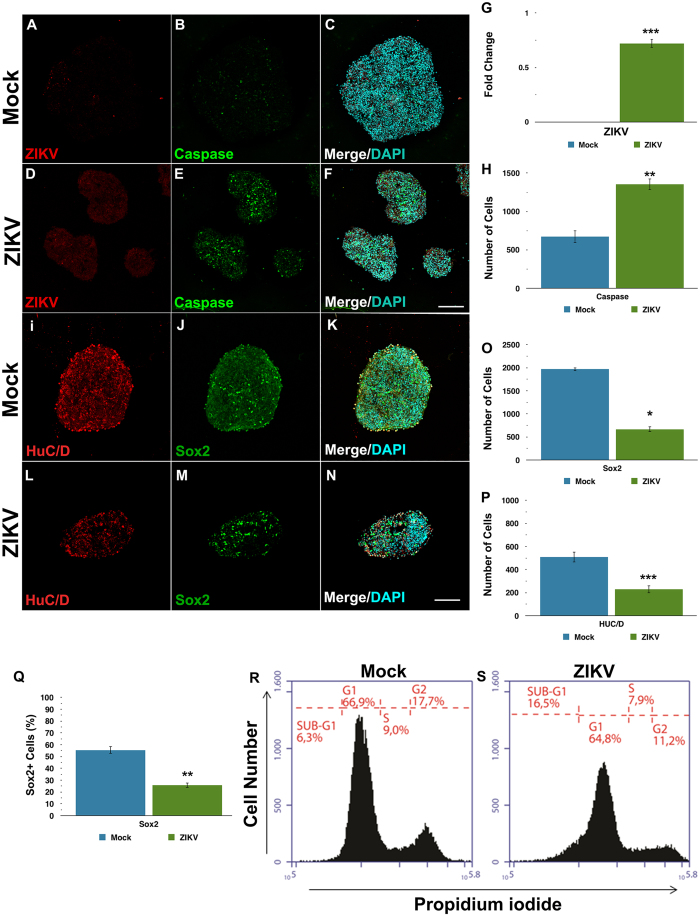
ZIKV reduces the growth of neurospheres, by depleting the pool of neural progenitors and the generation of neurons. (**A–F**) Immunocytochemistry for the flavivirus antigen (red) and cleaved caspase-3 (green) counterstained with DAPI (blue) on Mock- and ZIKV-infected neurospheres. (**G**) Quantification of flavivirus antigen and (**H**) cleaved caspase-3 fluorescence intensity. Individual sample data were normalized against the average of Mock-infected experimental group. (**I–P**) Immunocytochemistry for the neuronal marker HuC/D (red) and the neural progenitor marker SOX2 (green) counterstained with DAPI (blue). Quantification of HuC/D and SOX2 fluorescence intensity shows a decrease of both markers on ZIKV-infected neurospheres. (**Q,R**) Flow cytometry distribution analysis of neurospheres at subG1 phase of cell cycle 3 days after ZIKV or mock infection. Differences on bar graphs were expressed by fold change in relation to the average values of the Mock-infected group. Data presented as mean ± SD, n = 4, Student’s t-test, *p < 0.05; **p < 0.01; ***p < 0.001.

After three days of infection, viable neurospheres with ZIKV mediated growth impairments were further characterized. Immunostaining for markers of apoptosis, neural progenitors, and neuronal cell were employed. ZIKV-infected neurospheres showed increased levels of activated caspase 3 (Fig. 2A–H) and displayed higher amounts of pyknotic nuclei (Supplementary Fig. S7A,C) compared to mock. Additionally, the number of Nestin (Supplementary Fig. S7D–I) and SOX2 positive progenitor cells was reduced (Fig. 2J,K,M–O,Q). Neuronal markers MAP2 (Supplementary Fig. S7J–M) and Hu C/D were also decreased in the ZIKV infected group compared to mock (Fig. 2I,K,L,N,P). In addition to cell death, alterations in cell cycle contributed to a reduction in the number of neural progenitors and newborn neurons. Flow cytometry analyses revealed that cells in ZIKV-infected neurospheres accumulate abnormally in a sub-G1 phase (Fig. 2R,S). Altogether, these results suggest that iPS derived-human neural stem cells infected with ZIKV in neurospheres show a reduction in cell replication associated with abnormal cell cycle progression after three days *in vitro*.

Uncovering the molecular fingerprint of ZIKV infection is essential to understand the brain growth impairments caused by this virus. To determine the molecular consequences of ZIKV infection on neural stem cells in 3D culture, we extracted proteins and mRNA from mock and ZIKV-infected neurospheres after three days *in vitro*, and analyzed their proteome and transcriptome. We employed ultra-definition data-independent mass spectrometry (UDMS^E^), a state-of-the-art method in quantitative label-free shotgun proteomics^10^ and unbiased global transcriptome analysis (RNA-seq).

**Figure 3:**
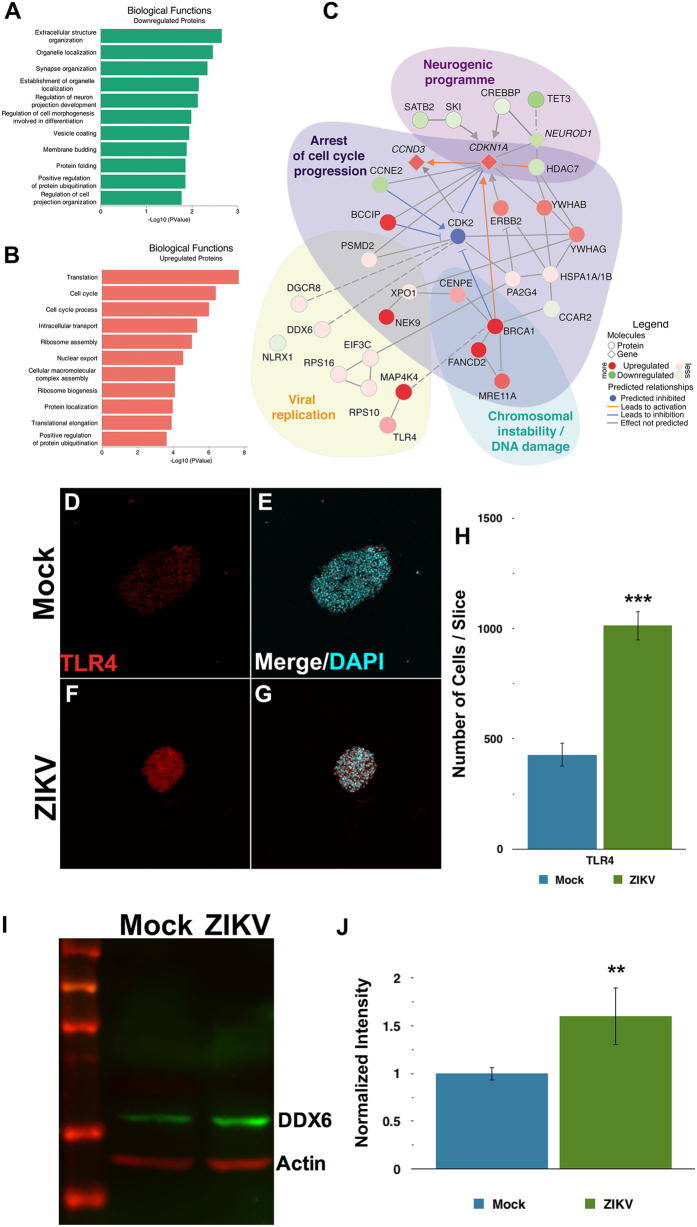
ZIKV alters translation, causing arrest of the cell cycle and decreasing neural differentiation in human neurospheres. Gene ontology enrichment of biological functions related to downregulated (**A**) and upregulated (**B**) proteins. (**C**) Network interactive representation of molecular relationship between regulated molecules on ZIKV-infected neurospheres, predicting downregulation of CDK2 and inhibition of cell cycle progress. Interactome analyzed from the dataset based on Ingenuity Knowledge Database (www.ingenuity.com) and String (string-db.org). As a validation of the mass spectrometry findings, we show (**D–H**) immunocytochemistry for TLR4 (red) counterstained with DAPI (blue), and (**I,J**) western blotting of DDX6, both upregulated on ZIKV-infected neurospheres compared to Mock-infected group. (**H**,**J**) Data presented as mean ± SD, Student’s t-test, **p < 0.01; ***p < 0.001.

We detected 199 downregulated and 259 upregulated proteins when comparing the proteomes of ZIKV-infected to mock-infected neurospheres (Supplementary Table S2). These deregulated proteins were categorized according to their gene ontology annotations (Fig. 3A,B and Supplementary Table S2). ZIKV-infected human neurospheres show downregulation of proteins involved in organelle localization, regulation, protein folding (Fig. 3A), and upregulation of translation and cell cycle related processes (Fig. 3B).

To gain further insight into the affected molecular pathways, we performed a regulation analysis that integrates RNA-Seq and proteomics data (Supplementary Table S3), to predict missing links to those altered pathways. Among the differentially expressed proteins are those required for viral replication, such as DDX6, eIF3c, and DGCR8 (Supplementary Fig. S8). Some of the targets identified by shotgun proteomics, such as TLR4 and DDX6 were validated using immunostaining or western blot. Both TLR4 and DDX6 are upregulated in ZIKV-infected neurospheres (Fig. 3D–J and Supplementary Table S2). Importantly, the interactome of differentially regulated proteins and genes in ZIKV-infected neurospheres (Fig. 3C) predict viral replication, cell cycle arrest, chromosomal instability and decline of the neurogenic program. Top upstream predicted regulators, EIF4 (p-value 6.86 × 10^−7^) and TP53 (p-value 1.20 × 10^−6^), are both linked to cell cycle and translation. Interestingly, we found BRCA1 (breast cancer type 1 susceptibility protein) and MRE11A (double-strand break repair protein) directly connected to those regulators. Both BRCA1 and MRE11A are related to DNA damage signaling or cell elimination if DNA is not repaired. As shown here, ZIKV-infected neurosphere cells have a pre-G1 enriched population, which is known to contain cells with damaged DNA^11^ (Fig. 2R,S). Similarly, the presence of pyknotic nuclei suggests that cell death is associated with DNA damage in ZIKV-infected neurospheres (Supplementary Fig. S7).

Taken together, our results suggest that ZIKV infection triggers the DNA repair machinery in human neural stem cells. Moreover, downregulation of Cyclin E (CCNE2) (Fig. 3C), in addition to upregulation of Cyclin-dependent kinase inhibitor 1A (CDKN1A) predicts prevention of the Cyclin E/CDK2 complex, a cell cycle progression regulator during the G1 phase. Therefore, these results point to a deregulated pathway that suggests how ZIKV infection can arrest the cell cycle. As a consequence, the neurogenic program is expected to be downregulated. Indeed, expression of transcription factors required for neuronal differentiation such as the pro-neural *NEUROD1* (neurogenic differentiation 1 gene) and SATB2 (Special AT-rich sequence-binding protein 2), were reduced in ZIKV-infected neurospheres.

## Discussion

Since 2015, ZIKV has been associated with primary microcephaly^2^,^3^. Previous studies described the effects of the African strain MR 766 in neural stem cells and brain organoids^4^,^5^,^6^. More recently, studies showed that both the Brazilian Zika virus and Asian strains were able to cause microcephaly in animal models *in vivo*^7^,^8^. However, the exact molecular pathways that produce brain abnormalities remain elusive. Here, we investigated the interactome of differentially regulated genes and proteins to gain further insights on how ZIKV causes growth defects before cell death. We applied proteomics and transcriptomics techniques and identified the molecular fingerprint of neurospheres infected by the ZIKV variant circulating in Brazil (Supplementary Fig. S9). The neurosphere is a three dimensional *in vitro* model that recapitulates early crucial processes of brain development such as progenitor expansion and neurogenesis, despite the absence of microglia, mature astrocytes, and endothelial cells.

Recent evidence points to radial glial cell progenitors as the major target of ZIKV in the developing brain^12^,^13^,^14^. Neurospheres are composed of neural progenitors at distinct stages of differentiation, including radial glia-like cells. Since ZIKV can affect different neural progenitors in distinct ways, we compared the exposure of ZIKV on two different progenitors: human iPS-derived neural stem cells and radial glia-like cells. ZIKV induced cell death in both neural progenitors with similar time frame and magnitude (Supplementary Figs S5 and S6), suggesting that the same targets are potentially affected by ZIKV in both cell types. Interestingly, it has been reported that neurons are not the major targets of ZIKV. Instead, glia seem to be preferential targets of this virus^4^,^13^, suggesting that cells with proliferation capabilities are preferred targets. Thus, despite the need for further studies to fully explore the differences of ZIKV infection on different neural cell types, describing the molecular fingerprint of ZIKV infection in neurospheres can shed light on identifying a broader range of targets and pathways affected by this virus.

Some of the genes differentially regulated by ZIKV had been previously found by the transcriptome analysis of infected human neural progenitors^4^,^15^,^16^. Upregulation of *CDKN1A* gene (cyclin-dependent kinase inhibitor 1A) is related to cell cycle arrest by preventing the phosphorylation of cyclin-dependent kinases. Others, such as SESN2 and GDF15, proteins involved in response to cellular stress after injury^17^,^18^ and downregulation of ARNT2, a bHLH factor required for neuronal differentiation^19^, are also commonly found in both studies^4^.

Nevertheless, our results identified several novel proteins and cellular pathways modulated by ZIKV in human neurospheres, that point to mechanisms of how ZIKV replicates at the expense of proliferation arrest and reduced production of neural cells. We showed that ZIKV upregulates a network of targets related to viral translation and DNA repair machinery, and downregulates molecules associated with cell cycle progression and neuronal differentiation. In summary, ZIKV infection modulates pathways such as RNA processing (DDX6, PCBP2), miRNA biogenesis (DGCR8, XPO1), translational initiation regulation (eIF3c), and proteins such as splicing factors (SFPQ, PRP8), ribosomal proteins (RPS6KA5, RPL28), innate immune response (TLR4) and neuronal development (*NEUROD1*, SATB2).

Some of the identified proteins differentially expressed in ZIKV infected cells are comparable to those induced by other RNA viruses. Many RNA viruses hijack cellular proteins to support viral replication, in particular, RNA binding proteins. Here we found that ZIKV increased DDX6 expression and translation (Fig. 3C,I,J and Supplementary Table S2). DDX6 (also known as RCK/p54) is an ATP-dependent RNA helicase in RNA processing bodies, which participate in translational suppression, mRNA degradation, and miRNA biogenesis. Upregulation of DDX6 has also been observed in HCV-related chronic hepatitis^20^. Downregulation of DDX6 impairs viral replication of DENV, HCV, and HCV RNA translation, strengthening its role in virus perpetuation^21^,^22^,^23^,^24^,^25^. Moreover, DDX6 has a pro-viral function in the assembly and release of infectious particles of DENV, that promotes virus replication^21^.

Viruses employ multiple strategies to deceive cells, delay intrinsic apoptotic signaling and allow viral replication cycles. Here we found that PRPF8 is upregulated by ZIKV infection (Supplementary Fig. S8 and Supplementary Table S2). PRPF8 is a splicing factor known to have an anti-apoptotic effect in neurons and Picornaviruses infection^26^,^27^. ZIKV infection also results in downregulation of a splicing factor, SFPQ, which might result in impairment of neuronal development. In this context, SFPQ is highly expressed in neural progenitors, and required for neuronal migration and differentiation^28^,^29^,^30^. Therefore, it remains to be shown how such differentially regulated targets are advantageous to the virus.

Furthermore, ZIKV infection triggers a cellular response by the innate immune system. Flavivirus RNA may be detected by RIG-I-like receptors (RLR) or Toll-like receptors (TLR), depending on RNA structure and cellular location. Infection of ZIKV in neurospheres upregulated the levels of TLR4, a receptor that mediates the innate immune response against other neurotropic flaviviruses, including Japanese Encephalitis virus^31^,^32^. Recently, it has been reported that ZIKV infection induces TLR3 expression in human skin fibroblasts^9^, human brain organoids^33^, and mouse embryos^8^. While TLR3 recognizes viral double-stranded RNA, TLR4 recognizes viral envelope glycoproteins^34^. Both receptors lead to pro-inflammatory cytokine production^35^. Modulation of the innate immune response should be considered in the search for therapeutic interventions against ZIKV.

Zika is now considered one of the STORCH factors, agents that cause intrauterine pathogenesis^36^. The Zika congenital syndrome is characterized by microcephaly, ventriculomegaly, periventricular calcifications, lissencephaly, agenesis of corpus callosum and other developmental abnormalities^37^. ZIKV infection of neural stem cells, brain organoids and mice brain development all lead to cell death^4^,^5^,^6^,^7^,^8^,^38^. Cell death could be the cause for the reduction of brain size, generating microcephaly. However, other mechanisms may also occur concomitantly with cell death upon ZIKV infection. For instance, the decreased neurosphere size could reflect an impairment in the ability of those cells to self-proliferate. According to previous microcephaly vera gene function studies, delays in the cell cycle reduces neuronal production^39^, and could account for the reduction in brain size. Interestingly, here we show that ZIKV alters cell cycle targets in progenitor cells, in parallel to cell death induction. Our work provides insights into the molecular mechanisms behind ZIKV self-replication, the arrest of cell cycle, downregulation of neurogenesis and increased cell death. It improves our understanding of the pathophysiology of ZIKV congenital syndrome and may contribute to the development of strategies to mitigate viral infection.

## Methods

### Ethics Statement

All protocols and procedures were approved by the institutional research ethics committee of Hospital Copa D’Or (CEPCOPADOR) under approved protocol # 727.269 and #1.269.816. ZIKV infected blood samples were obtained specifically for this study. The institutional research ethics committee of Hospital Copa D’Or (CEPCOPADOR) approved this protocol under #1.269.816. All adult subjects provided informed written consent, and a parent or guardian of any child participant provided informed written consent on their behalf. All experiments were performed in accordance with relevant guidelines and regulations.

### Human induced pluripotent stem cells

Human induced pluripotent stem cells (iPS) were provided by the Biobank of iPS cells of the Brazilian Ministry of Health (CONEP B-027 # 25000.111598/2014-04). According to the supplier, fibroblasts were reprogrammed using the protocol developed by Paulsen *et al*., 2012^40^, transduced with the CytoTune^®^-iPS Sendai kit. iPS cells presented a normal karyotype and the expression of pluripotency markers. iPS cells were cultured either with Essential 8 medium (Thermo Fisher Scientific, USA) containing DMEM/F12 and supplement, or mTeSR1 (Stemcell Technologies, USA) on Matrigel (BD Biosciences, USA) coated dishes. The colonies were chemically passaged (0.5 mM EDTA, Thermo Fisher Scientific, USA) every 5–7 days until they reached 70–80% confluence and maintained at 37 °C in humidified air with 5% CO_2_.

### Human neural stem cells and glial cells

Human induced pluripotent stem cells were cultured either with Essential 8 medium (Thermo Fisher Scientific, USA) containing DMEM/F12 and supplement, or mTeSR1 (Stemcell Technologies, USA), on Matrigel (BD Biosciences, USA) coated surface. The colonies were manually passaged every 5–7 days until they reached 70–80% confluence and maintained at 37 °C in humidified air with 5% CO_2_. To induce differentiation to neural stem cells (NSCs), human iPS cells were split and 24 hours later medium was switched to PSC neural Induction Medium (Thermo Fisher Scientific, USA), containing Neurobasal medium and PSC supplement, according to manufacturer’s protocol. Media was changed every other day until day 7, during which neural stem cells were split and expanded on neural induction medium (Advanced DMEM/F12 and Neurobasal medium (1:1) with neural induction supplement; Thermo Fisher Scientific, USA). NSCs were differentiated into glial cells as described in Yan, 2013^41^. Briefly, NSCs were placed in differentiation media (1% N2 supplement and 1% FBS in DMEM/F12) for 21 days with media changes every other day and passages every week. After this period, glial cells were kept in 10% FBS in DMEM/F12 with media changes after 2–3 days for 2 weeks.

### Human neurospheres

Neural stem cells were cultured until 80% confluence and split with Accutase (Merck-Millipore, Germany). Cells were resuspended in neural medium with half DMEM/F12 and half Neurobasal medium supplemented with 1X N2 and 1X B27 supplements. The suspended neural stem cells were grown under rotation at 90 rpm, and media was replaced every 4 days. Images were acquired using the EVOS Cell Imaging System (Thermo Fisher Scientific, USA). Neurosphere area was measured with ImageJ (μm^2^).

### Infection of neural stem cells with ZIKV

ZIKV was isolated from the blood of a patient from Brazil. The virus was propagated in Vero cells (ATCC #CCL-81). Neural stem cells were either in the mock or ZIKV-infected group/condition. At 96 hours post-infection (hpi), cytopathic effects were observed and conditioned media from mock and ZIKV-infected cells was harvested, centrifuged at 300 × g and stored at −80 °C. ZIKV genome was sequenced and strain identity was confirmed (accession number NC_012532). ZIKV titers were determined by a plaque assay performed in Vero cells. Neural stem cells were ZIKV-infected at MOIs ranging MOI ranging from 0.25 to 0.0025. Cells were incubated with virus or mock for 2 hours, after which media containing virus particles was replaced with fresh media.

### RNA extraction and RT-PCR

ZIKV stock sample was analyzed by RT-PCR to detect Zika, Chikungunya and Dengue viruses. Briefly, viral RNA was extracted using QIAamp MinElute Virus Spin Kit (Qiagen, Netherlands) and cDNA synthesis was performed with High-Capacity cDNA Reverse Transcription Kit (Thermo Fisher Scientific, USA) according to the manufacturer’s instructions. RT-PCR was performed using TaqMan Universal Master Mix (Thermo Fisher Scientific, USA) on 7500 Real-Time PCR System (Applied Biosystems) with primers and probes specifically designed for ZIKV^42^, CHIKV^43^ and DENV^44^.

### Immunostaining

Neurospheres were fixed with 4% paraformaldehyde (Sigma-Aldrich, USA) in phosphate-buffered saline for 15 min at 37 °C, cryoprotected in sucrose solution, mounted in OCT embedding compound and frozen at −20 °C. Thin sections (20 μm) were obtained with a cryostat (Leica, Germany), permeabilized with 0.3% Triton X-100 (Sigma-Aldrich, USA), incubated in 50 mM ammonium chloride, followed by 3% bovine serum albumin blocking (Sigma-Aldrich, USA). Incubation with rabbit anti-human-Sox2 (1:100; Merck-Millipore, Germany), rabbit anti-human-caspase 3 active (cleaved) form (1:100; Merck-Millipore, Germany), rabbit anti-GFAP (1:100); mouse anti-human-HuC/HuD neuronal protein (16A11) (1:100, Invitrogen, USA), mouse anti-H2aX (1:100), mouse anti-flavivirus group antigen antibody (clone 4G2, 1:100), rabbit anti-PAX6 (1:100, Santa Cruz Biotechnologies, USA) mouse anti-TLR4 (H-80) (1:100, Santa Cruz, USA), rabbit anti-Vimentin (1:500, abcam, USA) and mouse anti-phospho (S55) Vimentin (1:250, abcam, USA) was performed overnight. Subsequently, samples were incubated with secondary antibodies: goat anti-rabbit Alexa Fluor 488 IgG (1:400; Thermo Fisher Scientific, USA) and goat anti-mouse Alexa Fluor 594 IgG (1:400; Thermo Fisher Scientific, USA). Nuclei were stained with 0.5 μg/mL 4′-6- diamino-2-phenylindole (DAPI) for 5 min. Images of three neurosphere slices of each condition were acquired with TCS SP8 confocal microscope (Leica, Germany) with an oil immersion 20x objective, (0.75 NA). Image analysis was performed by applying automatic segmentation and counting methods for each marker using image analysis platform Columbus (PerkinElmer, USA) as follows. For each histological section of neurosphere, we counted the total number of cells labeled with Hu C/D, Sox2 or Caspase-3 and normalized by the total number of DAPI nuclei. The individual level of DAPI staining is not be affected by viral infection. This quantification was done in three independent experiments, with more than 20 different neurospheres. Results are presented as an average of neurospheres per condition.

### Cresyl Violet staining and pyknotic nuclei analyses

Neurospheres were sectioned and stained with cresyl violet. Photomicrographs were taken with a magnification of 400X on an Axioplan Zeiss microscope. Cells displaying condensed pyknotic and fragmented nuclei were identified, counted under ImageJ 2.0 (NIH) and grouped in the category “pyknotic nuclei”. Percentage of pyknotic nuclei was determined by normalization by total cell number.

### Shotgun Proteomics and Data Analyses

Qualitative and quantitative proteomic analyses were performed in a two dimensional nano UPLC (2D-RP/RP) in a Acquity UPLC M-Class System (Waters Corporation, USA), which is connected in line to a Synapt G2-Si mass spectrometer (Waters Corporation, USA). The mass spectrometer executed Data-Independent Acquisitions (DIA), employing more specifically Ultra-definition Data-independent Mass Spectrometry (UDMS^E^) method^10^. Peptide loads were carried to separation in a nanoACQUITY UPLC HSS T3 Column (1.8 μm, 75 μm × 150 mm; Waters Corporation, USA). Peptide elution was achieved using an acetonitrile gradient from 7% to 40% (v/v) for 95 min at a flow rate of 0.4 μL/min directly into a Synapt G2-Si. For every measurement, the mass spectrometer was operated in resolution mode with an m/z resolving power of about 40 000 FWHM, using ion mobility with a cross-section resolving power at least 40 Ω/ΔΩ. MS/MS analyses were performed by nano-electrospray ionization in positive ion mode nanoESI (+) and a NanoLock Spray (Waters, UK) ionization source. The lock mass channel was sampled every 30 sec. The mass spectrometer was calibrated with an MS/MS spectrum of [Glu1]-Fibrinopeptide B human (Glu- Fib) solution that was delivered through the reference sprayer of the NanoLock Spray source. Proteins were identified and quantitative data were processed by using dedicated algorithms and cross-matched with the Uniprot human proteome database, version 2015/11 (70,225 entries), with the default parameters for ion accounting and quantitation^45^. The databases used were reversed on-the-fly during the database queries and appended to the original database to assess the false- positive identification rate. For proper spectra processing and database searching conditions, we used Progenesis QI for proteomics software package with Apex3D, peptide 3D, and ion accounting informatics (Waters). This software starts with LC- MS data loading, then performs alignment and peak detection, which creates a list of interesting peptide ions (peptides) that are explored within Peptide Ion Stats by multivariate statistical methods; the final step is protein identity. The following parameters were considered in identifying peptides: (1) Digestion by trypsin allowing one missed cleavage; (2) methionine oxidation was considered a variable modification and carbamidomethylation (C), fixed modification; (3) false discovery rate (FDR) less than 1%. Identifications that did not satisfy these criteria were rejected. Raw mass spectrometry (MS) files used in this experiment have been uploaded to the proteomics data repository PRIDE with the accession number PXD004588.

### Pathway and functional correlation analysis

Differentially expressed genes and proteins were analyzed. Functional annotation analysis tool DAVID v 6.7 (http://niaid.abcc.ncifcrf.gov) was used to identify over-represented ontological groups amongst differentially expressed proteins of mock and ZIKV-infected neurospheres and to group proteins into functional categories^46^,^47^. Whole genome was used as a background list. The overrepresented GO terms (GOTERM_ALL level) using default settings and p ≤ 0.01 under Fisher exact test. Ingenuity^®^ Pathway Analysis (IPA^®^, QIAGEN Redwood City, USA, www.qiagen.com/ingenuity), was used to identify proteins and genes that may be part of pathway dysregulation caused by ZIKV. IPA^®^ core analysis was performed using experimentally observed data, with canonical pathways, diseases and biological functions, and networks explored in detail. The refinement of the network generated by IPA^®^ was performed applying the following parameters: direct/indirect interactions, experimentally observed as confidence level, human as species. In addition, we explored the protein-protein and gene-protein interactome using most relevant biological functions, as an interactive representation that shows the molecular relationship between molecules from the dataset based on Ingenuity Knowledge Database. In addition, protein-protein regulatory network information from high confidence STRING interactions (www.string-db.org) was added to this interactome^48^.

### Western blotting

Cell lysate of ZIKV and mock infected-neurospheres were used for estern blot analysis with RCK (1:500, sc-51415, Santa Cruz Biotechnology, Inc., Dallas, TX, USA) and actin antibodies (1:1000, MAB1501, Merck Millipore Corporation, Darmstadt, Germany). IRDye^®^ secondary antibodies were used followed by Odyssey^®^ Imaging System detection (LI-COR Biotechnology, Inc., Lincoln, NE, USA). Blots were normalized to actin as a loading control. Two independent experiments in duplicate were blotted.

### RNA-Seq

Total RNA isolation from both conditions of neurospheres (MOCK and ZIKV infection) was done with iPrep PureLink Total RNA Kit (Thermo Fisher Scientific, USA). For total RNA quantitation and quality analyses we used Qubit RNA HS Assay Kit (Thermo Fisher Scientific, USA) and Agilent RNA 6000 Pico Kit (Agilent Technologies, USA). RNA-seq was performed using duplicated samples for each condition; libraries were built using TruSeq Stranded mRNA Library Prep Kits (Illumina, USA) according manufacturer’s instructions. To evaluate fragment average and quantify libraries we used Agilent 2100 BioAnalyzer and High Sensitivity DNA Kit (Agilent Technologies, USA) and a qPCR-based KAPA library quantification kit (KAPA Biosystems, USA), finally we pooled libraries with 20 pM of final concentration to following sequencing steps. For paired-end sequencing we used MiSeq Sequencing System (Illumina, USA) platform and MiSeq Reagent Kit v3 (150 cycles). Illumina reads underwent quality control analysis using FastQC (http://www.bioinformatics.babraham.ac.uk/projects/fastqc/). The reads were aligned with TopHat version 2.1.1^49^ using the EMBL human genome GRCh37 as reference. Aligned reads were assembled de novo using CuffLinks version 2.2.1^50^. FPKM (Fragments Per Kilobase of transcript per Million reads mapped) values, which reflect mRNA expression levels, were generated using CuffDiff that is part of CuffLinks software. The R package CummeRbund^51^ was used to visualize the results. Ingenuity Pathway Analysis (www.ingenuity.com; Ingenuity Systems, Redwood City, CA, USA) was employed to identify biological functions associated with the differentially expressed genes.

### Viral RNA isolation and genome sequencing

First, viral RNA was isolated from infected neurospheres using iPrep PureLink Virus Kit (Thermo Fisher Scientific) and the cDNA synthesis reaction was performed using cDNA Synthesis System Kit (Roche), with random primers. After, PCR was done using specific primers to Asian ZIKV genotype sequence to get amplicons for sequencing. Amplicons were fragmented by enzymatic digestion and libraries built by the automated AB Library Builder System (Applied Biosystems). Libraries were submitted to emulsion PCR using the automated Ion OneTouch 2 platform. The emulsion PCR reaction was loaded on a 318v2 chip and the sequencing reaction was performed on the Ion PGM™ (Thermo Fisher Scientific, USA).

### Genome assembly and phylogenetic analysis

Genome assembly was performed using Geneious v.9.1.3 using the genome of a Zika virus strain isolated in Brazil as reference (accession number KU926310). Minimum overlap and minimum overlap Identity parameter values were 40 and 95%, respectively. We obtained a partial sequence including partial envelope, NS1, NS2A, NS2B and NS3 from ZIKV genome. The obtained genome from BR_ZIKV_AB_ES isolate was deposited in GenBank with accession number KX212103. The genomic sequence of BR_ZIKV_AB_ES was aligned with other ZIKV sequences available in NCBI using Mafft v.7^52^. Measurement of phylogenetic signal in the aligned dataset was done using likelihood mapping test, as employed in Tree-Puzzle v 5.2^53^. The analysis revealed only 17% unresolved and 2.8% partly resolved quartets, indicating that the dataset has a strong phylogenetic signal. The set of aligned data was submitted to jModelTest v.1.2.7^54^, in order to find the best nucleotide substitution model. The construction of the phylogenetic tree was performed with maximum likelihood (ML) method^55^ using RaxML v.8.0^56^. A bootstrap test with 1,000 replicates was applied to provide confidence values^57^ (Supplementary Fig. 1A). Genetic distances amongst strains were calculated using MEGA 6.06^58^. The evolutionary divergence between ZIKV isolated sequences was analyzed using Geneious v9 (www.geneious.com 59).

## Additional Information

**How to cite this article**: Garcez, P. P. *et al*. Zika virus disrupts molecular fingerprinting of human neurospheres. *Sci. Rep.* **7**, 40780; doi: 10.1038/srep40780 (2017).

**Publisher's note:** Springer Nature remains neutral with regard to jurisdictional claims in published maps and institutional affiliations.

## Supplementary Material

Supplementary Information

Supplementary Table S1

Supplementary Table S2

Supplementary Table S3
