# Supplementary material for: Zika virus disrupts molecular fingerprinting of human neurospheres
Source: Sci Rep. 2017 Jan 23;7:40780. doi: 10.1038/srep40780 (PMC5256095; doi:10.1038/srep40780)
Supplement: Supplementary Information [file srep40780-s1.pdf]

Supplementary information

Zika virus disrupts molecular fingerprinting of human neurospheres

Patricia P. Garcez[2,1@], Juliana Minardi Nascimento[1,3], Janaina Mota de Vasconcelos[4], Rodrigo Madeiro da Costa[1], Rodrigo Delvecchio[5], Pablo Trindade[1], Erick Correia Loiola[1], Luiza M. Higa[5], Juliana S. Cassoli[3], Gabriela Vitória[1], Patricia Sequeira[6], Jaroslaw Sochacki[1], Renato S. Aguiar[5], Hellen Thais Fuzii[7], Ana M. Bispo de Filippis[6], João Lídio da Silva Gonçalves Vianez Júnior[4], Amilcar Tanuri[5], Daniel Martins-de-Souza[3@], Stevens K. Rehen[1, 2 @]

[@]Corresponding authors: srehen@lance.ufrj.org, ppgarcez@icb.ufrj.br, and dmsouza@uni-camp.br

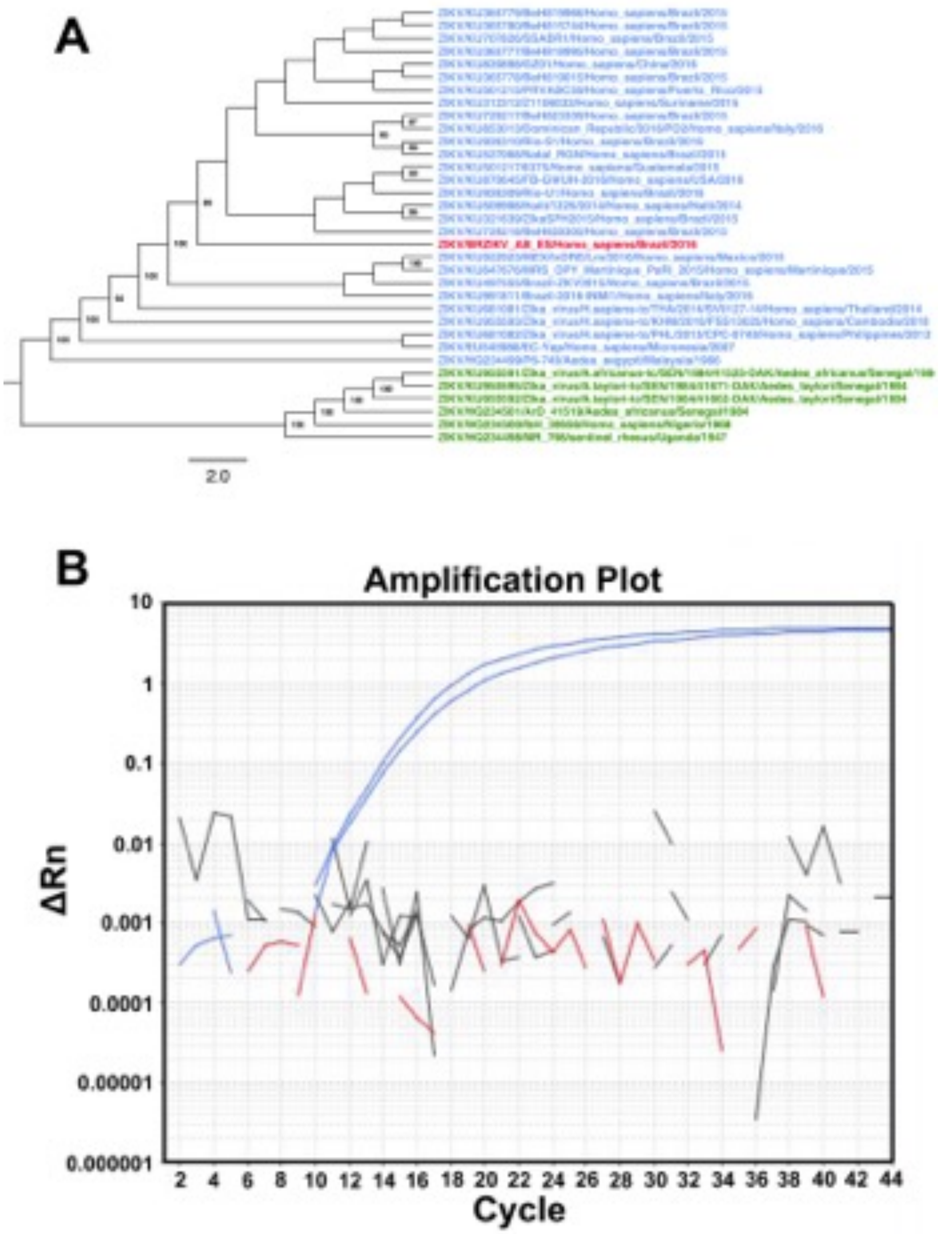

Supplementary Figure S1: Asian genotype of ZIKV isolated in Brazil

(A) Phylogenetic analysis including BRZIKV_AB_ES isolate and ZIKV available sequences. Both genotypes are represented on this ML (*maximum likelihood*) tree: in blue, Asian, in green, African genotype and, highlighted in red, the partial ZIKV sequence obtained from infected neurospheres here described (BRZIKV_AB_ES). The bootstrap values higher than 85% are shown. This analysis demonstrates that the isolated Brazilian ZIKV strain belongs to Asian genotype. (B) ZIKV stock sample was analyzed by RT-PCR to detect Zika (blue lines), chikungunya (red line) and Dengue (black line) viruses.

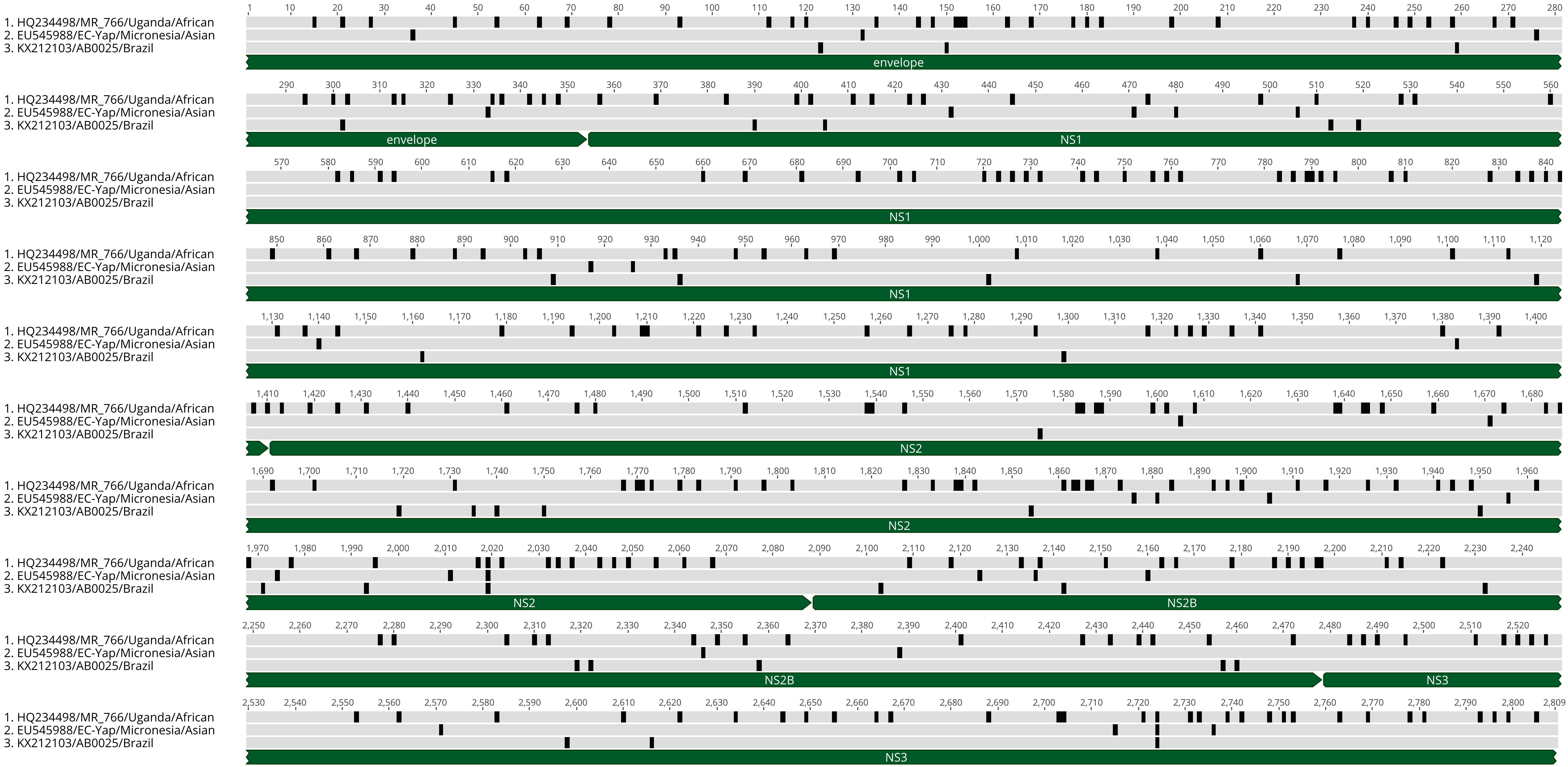
1. HQ234498/MR_766/Uganda/African
2. EU545988/EC-Yap/Micronesia/Asian
3. KX212103/AB0025/Brazil
envelope
NS1
NS2
NS2B
NS3

Supplementary Figure S2: Genome map showing the conservation among the genes from an African (HQ234498), Asian (EU545988) strains and BRZIKV_AB_ES (KX212103).

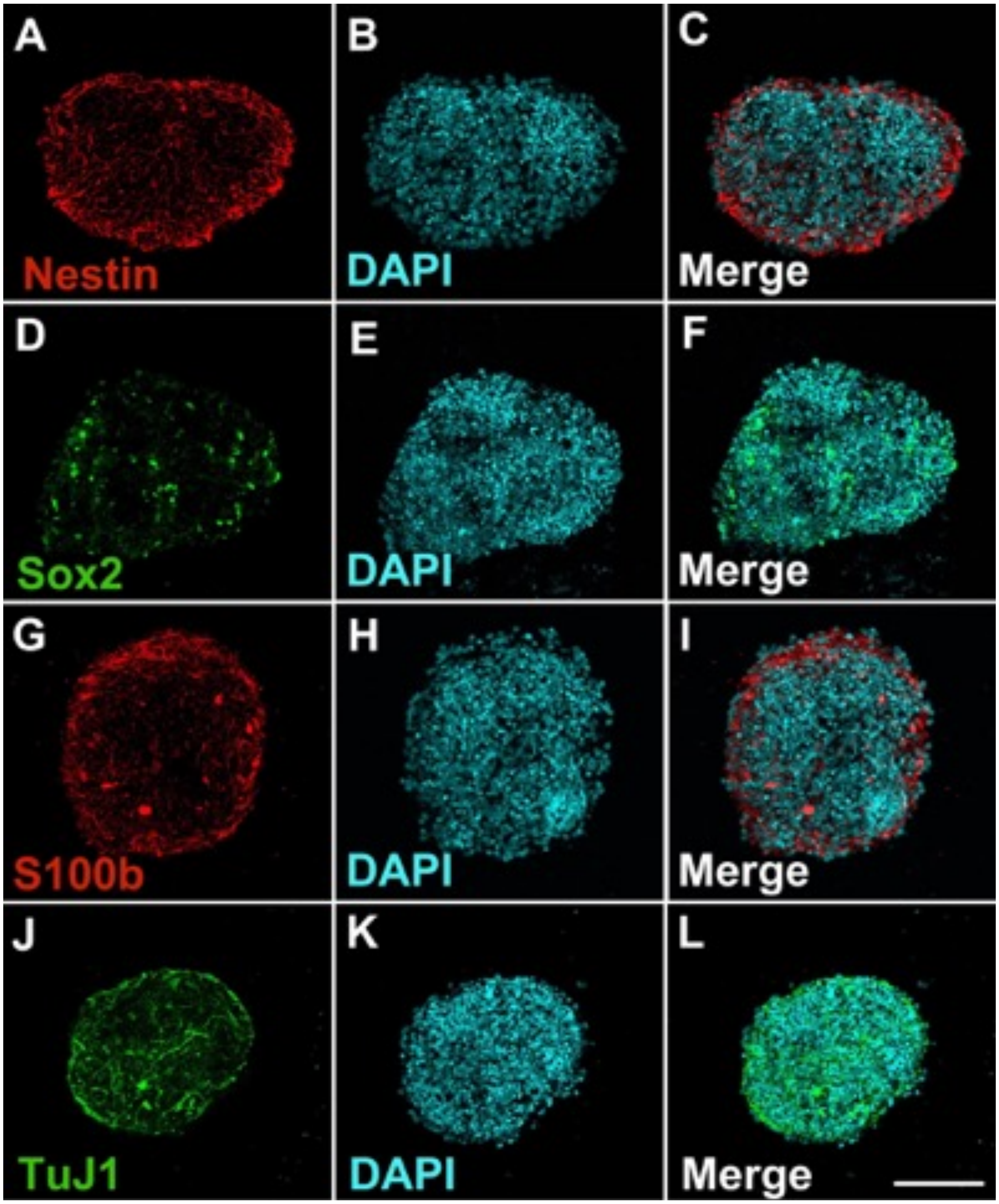

Supplementary Figure S3: After 3 days i*n vitro* neurospheres express neural progenitor (Nestin, Sox2, S100b) and neuronal markers (TuJ1). Calibration Bar: 100µm

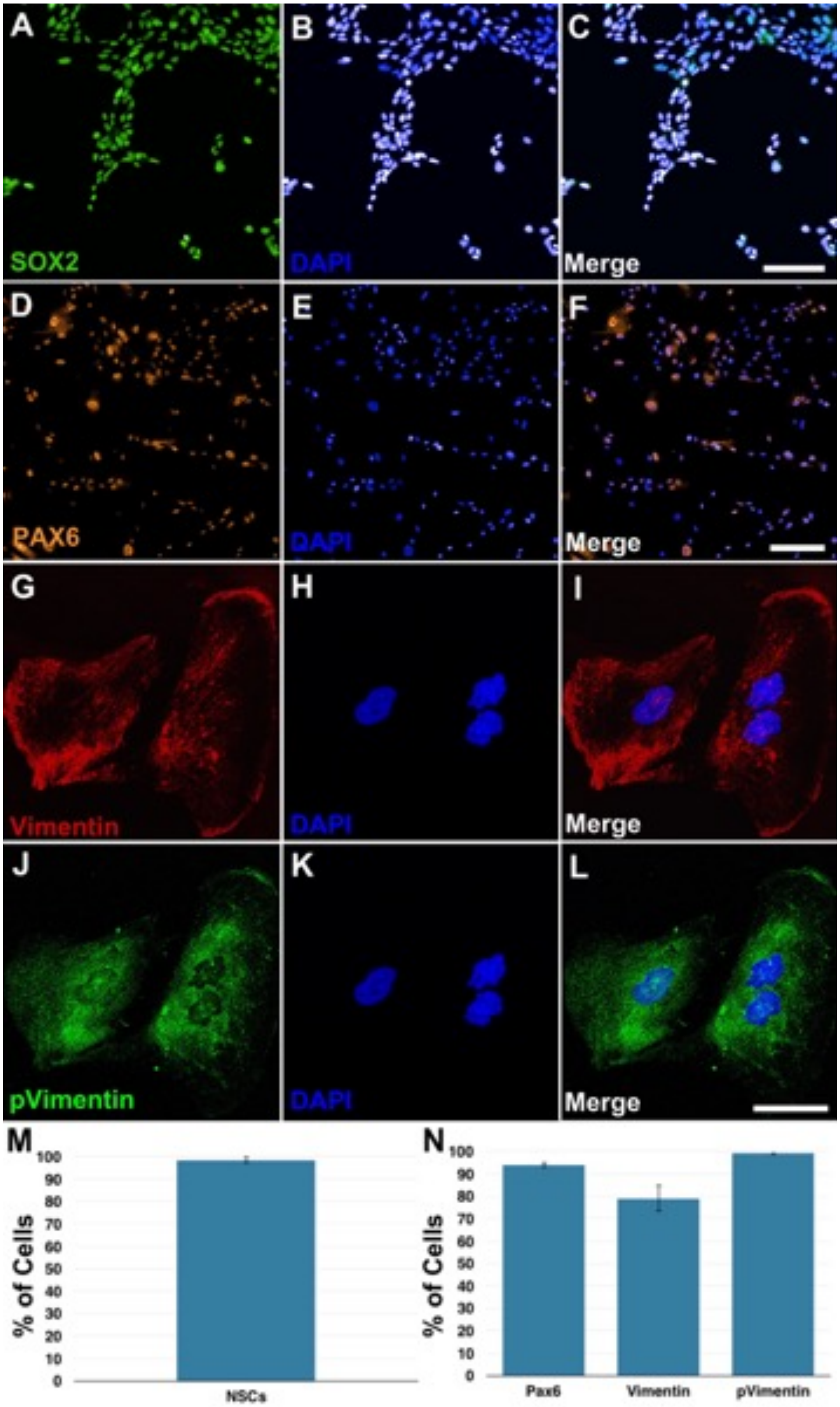
A
SOX2
B
DAPI
C
Merge
D
PAX6
E
DAPI
F
Merge
G
Vimentin
H
DAPI
I
Merge
J
pVimentin
K
DAPI
L
Merge
M
% of Cells
NSCs
N
% of Cells
Pax6
Vimentin
pVimentin

Supplementary Figure S4. Immunocytochemistry for neural progenitor markers on human iPSs-derived neural stem cells (NSCs) and radial glia-like cells (RGCs). (A-C), virtually all NSCs were found positive for Sox2. Calibration bar on C = 100 μm. (D-F) Pax6 labeling was found in the majority of RGCs. Calibration bar on F = 200 μm. **G-I**, Vimentin labeling was found more abundantly on extremities of RGCs. (J-L) phosphorylated Vimentin (pVimentin) was found widespread in the cell body of all RGCs. Calibration bar on L (refered to G-L) = 50 μm. (M) quantification of number of NSCs positive for Sox2. (N) quantification of number of RGCs positive for Pax6, Vimentin and pVimentin.

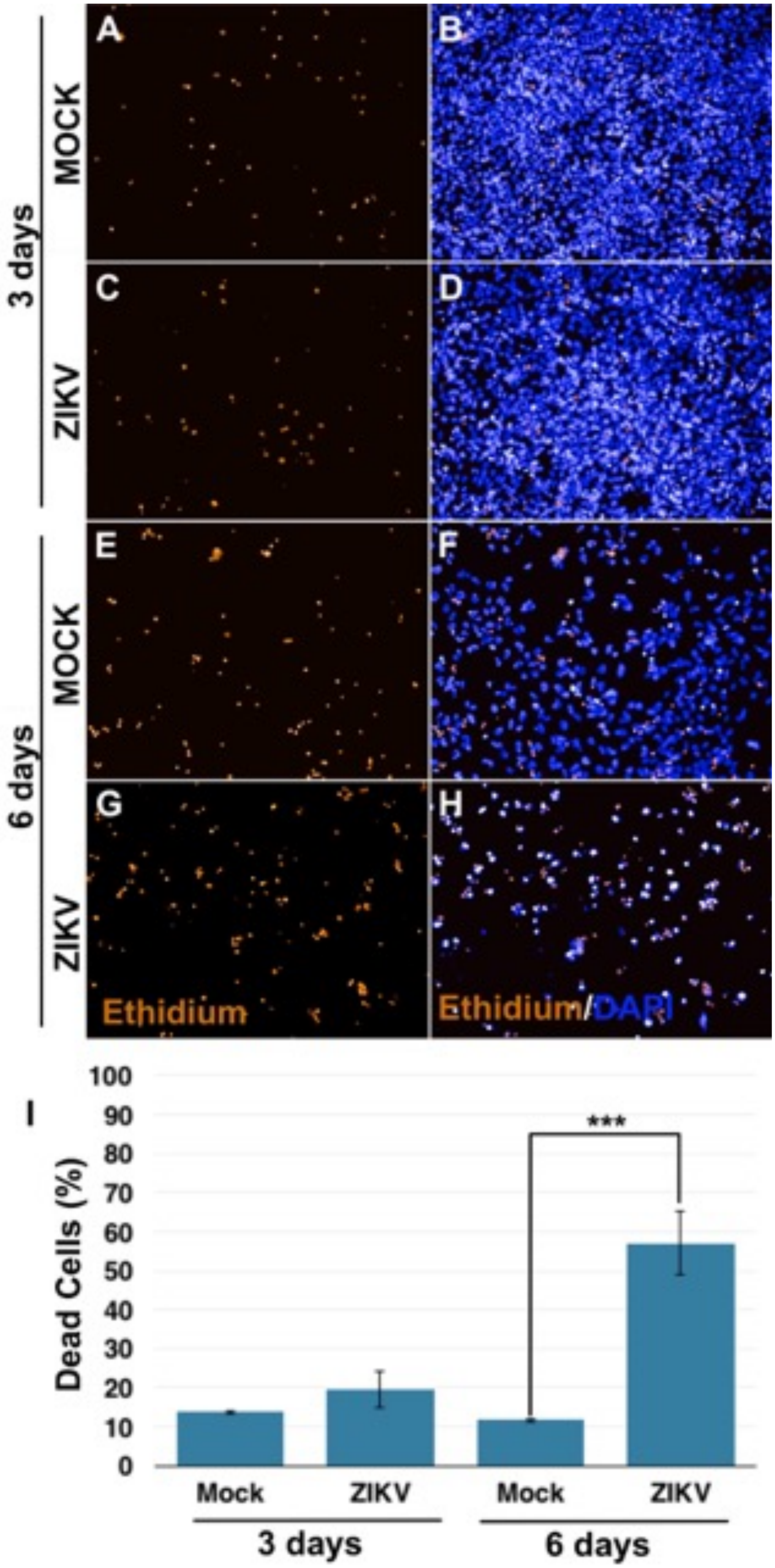
3 days
6 days
MOCK
ZIKV
A
B
C
D
E
F
G
H
Ethidium
Ethidium/DAPI
I
Dead Cells (%)
100
90
80
70
60
50
40
30
20
10
0
***
Mock
ZIKV
Mock
ZIKV
3 days
6 days

Supplementary Figure S5. Ethidium incorporation labeling following ZIKV infection on NSCs. (A-B**)**, ethidium incorporation on mock-infected cells after 3 days of infection. (C-D) ZIKV-infected NSCs do not show strong ethidium incorporation following 3 days of infection. (E-F) ethidium incorporation on mock-infected cells after 6 days. (G-H) Many ZIKV-infected NSCs died following 6 days of infection and detached from the plate. Most of the cells that left were found labeled with ethidium. (**I**) quantification of dead cells by ethidium incorporation. ***$p<0.001$.

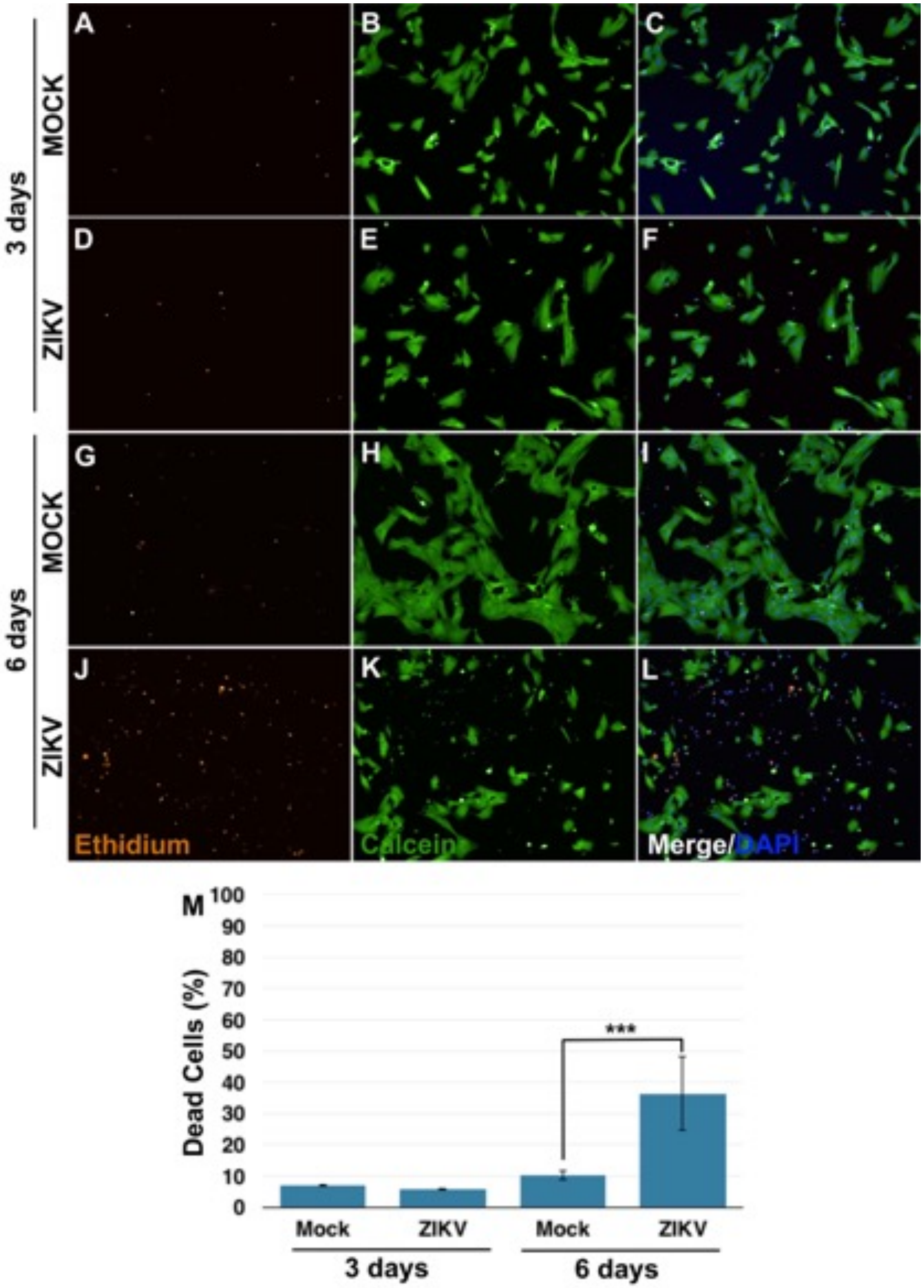
3 days
6 days
MOCK
ZIKV
MOCK
ZIKV
A
B
C
D
E
F
G
H
I
J
K
L
Ethidium
Calcein
Merge/DAPI
M
Dead Cells (%)
100
90
80
70
60
50
40
30
20
10
0
***
Mock
ZIKV
Mock
ZIKV
3 days
6 days

Supplementary Figure S6. Ethidium and calcein incorporation labeling following ZIKV infection on RGCs. (A-C) ethidium and calcein incorporation on mock-infected cells after 3 days of infection. D-F, ZIKV infection do not affect ethidium incorporation following 3 days of infection on RGCs. (G-I) ethidium and calcein incorporation on mock-infected RGCs after 6 days. (J-L) Many ZIKV-infected RGCs died following 6 days of infection or exhibited a shorter cell body area. (M) quantification of dead cells by ethidium incorporation. ***$p<0.001$.

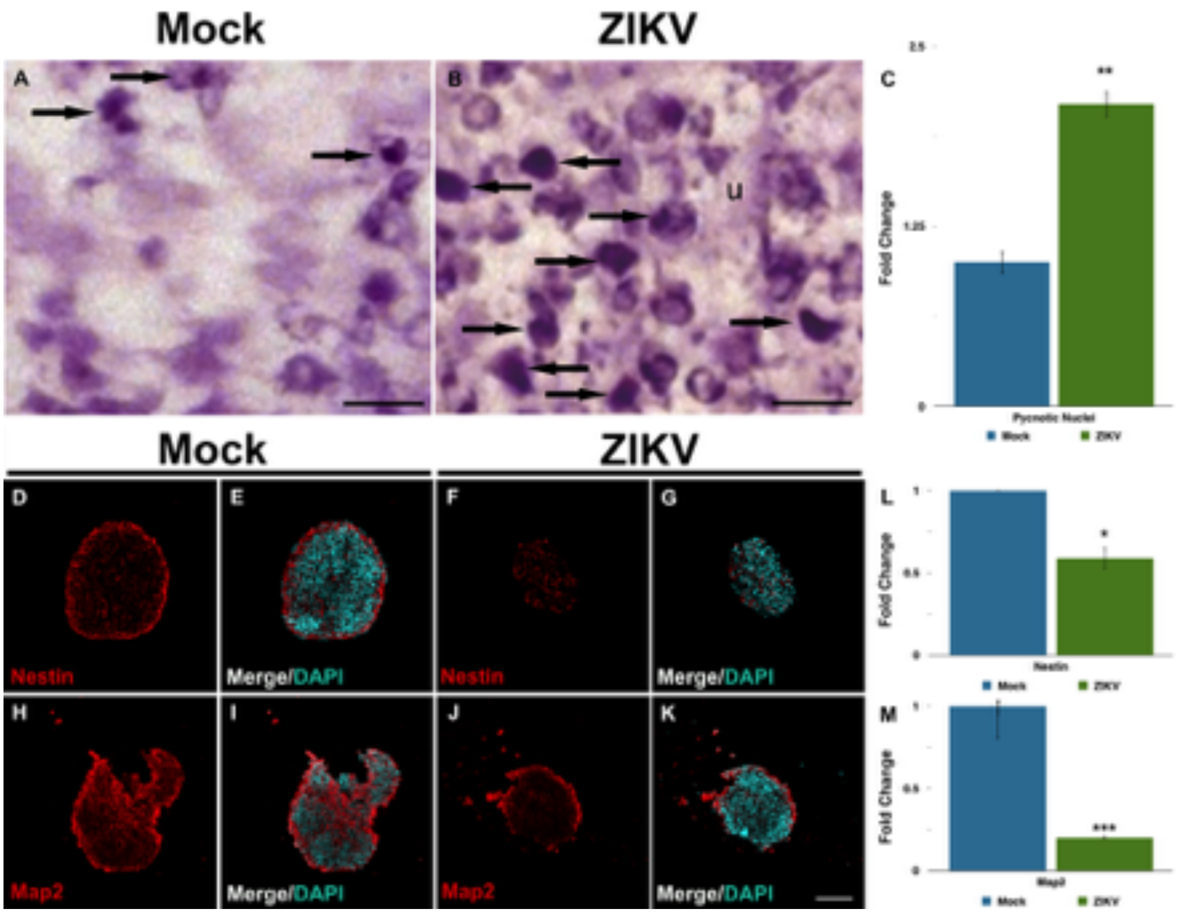

Supplementary Figure S7: ZIKV infection increases the number of cells displaying pyknosis and decreases progenitors and neuronal markers on neurospheres

(A-B) Brightfield photomicrographs of cells displaying condensed pyknotic (arrows) and fragmented nuclei (arrowheads). Calibration Bar: 10µm. (C) Quantification of the percentage of pyknotic nuclei found on Mock- and ZIKV- infected neurospheres. Data presented as mean ± SEM, n=4, Student's t-test, ** p<0.01. Immunocytochemistry for the progenitor marker nestin (red) on mock- (D-E) and on ZIKV-infected neurospheres (F-G) counterstained with DAPI (blue). (H) Quantification of neural progenitor marker nestin fluorescence intensity. Immunocytochemistry for the neuronal marker MAP2 (red) on mock- (I-J) and ZIKV-infected neurospheres

(K-L) counterstained with DAPI (blue). (M) Quantification of neuronal marker MAP2 fluorescence intensity. Data presented as mean ± SD, n=4, Student's t-test, *$p < 0.05$. ; *** $p<0.001$ Calibration Bar: 100μm

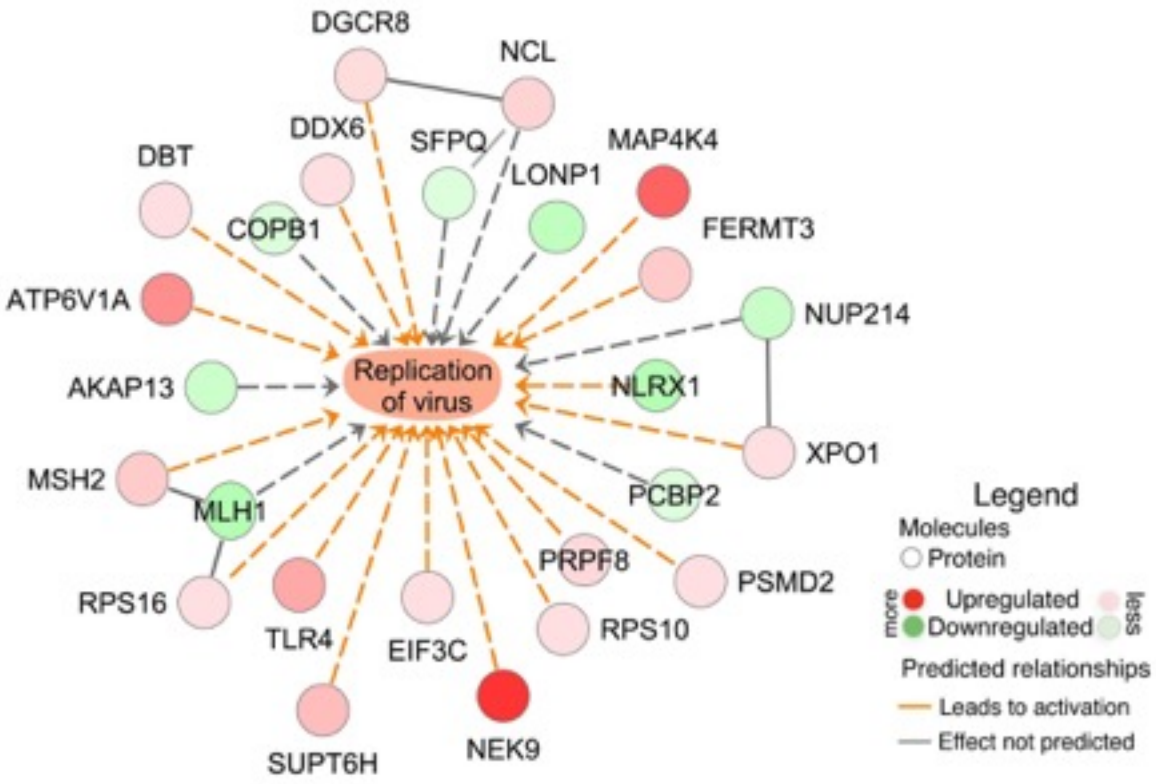

Supplementary Figure S8: ZIKV infection upregulates proteins required for viral replication. Network interactive representation of molecular relationship between regulated molecules on ZIKV-infected neurospheres. Interactome analyzed from the dataset based on Ingenuity Knowledge Database (www.ingenuity.com) and String (string- db.org).

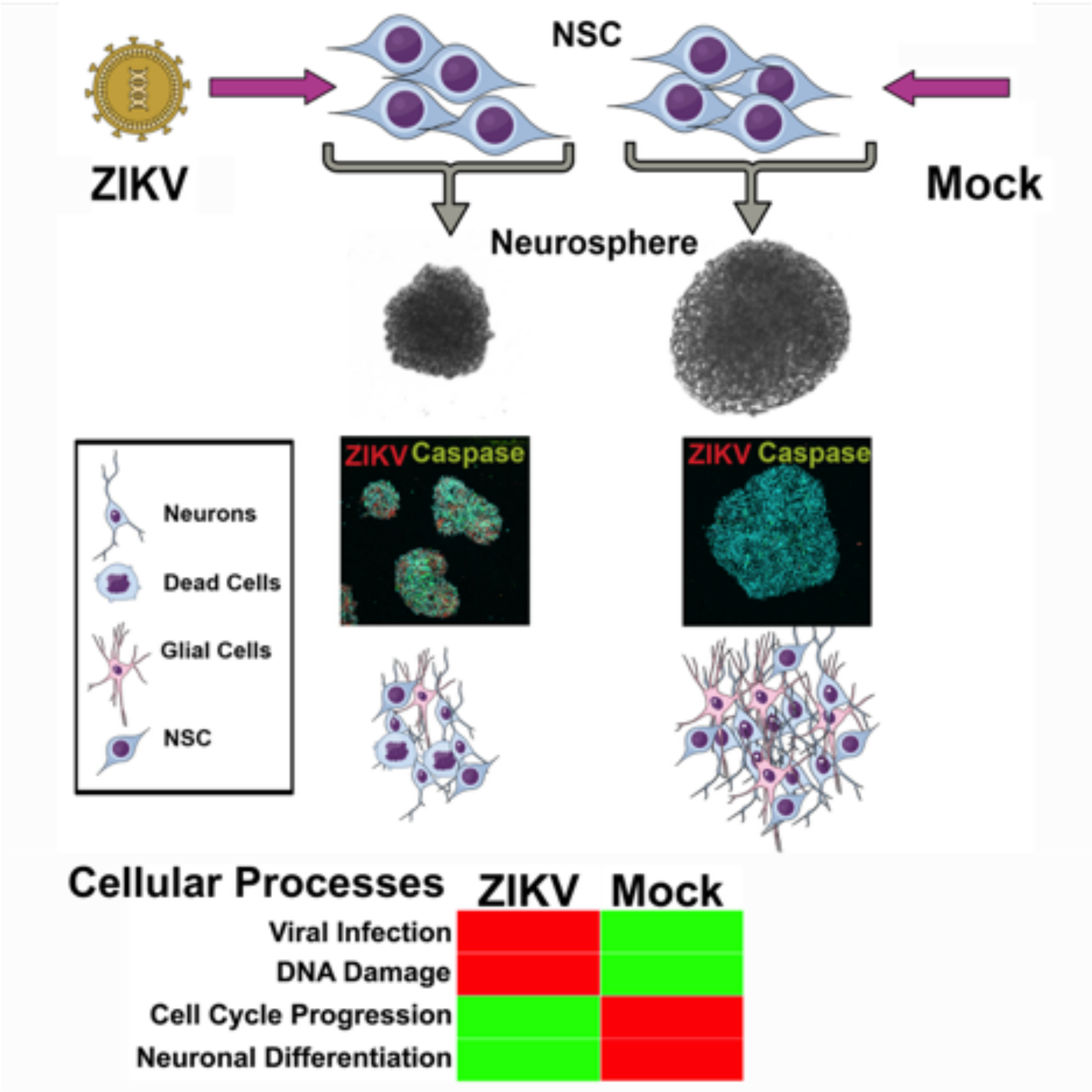

Supplementary Figure S9: Summary of the molecular fingerprint of human neural cells infected by the ZIKV variant circulating in Brazil. Figure created in the Mind the Graph platform, (www.mindthegraph.com). Licensed under the Attribution-ShareAlike 4.0 International license. The license terms can be found on the following link: https://creativecommons.org/licenses/by-sa/4.0/.

Supplementary Table S1:

Genetic analysis of evolutionary divergence between ZIKV isolated sequences. The nucleotide base per site was compared between sequences with the software Geneious v 9 (www.geneious.com).

Supplementary Table S2:

Differential protein expression between ZIKV- and mock-infected human neurospheres.

Supplementary Table S3:

Differential gene expression between ZIKV- and mock-infected human neurospheres.
